# Supplementary material for: Two-stage penalized regression screening to detect biomarker-treatment interactions in randomized clinical trials
Source: Biometrics. Author manuscript; Available in PMC 2022 Nov 27. (PMC7613856; doi:10.1111/biom.13424)
Supplement: Supplementary File [file EMS157113-supplement-Supplementary_File.zip › biom13424-sup-0001-SuppMat.pdf]

BIOMETRICS 000, 000–000 DOI: 000
000 0000

Supporting Information for "Two-Stage Penalized Regression Screening to Detect Biomarker-Treatment Interactions in Randomized Clinical Trials" by Jixiong Wang, Ashish Patel, James M.S. Wason, and Paul J. Newcombe

Jixiong Wang[1,*], Ashish Patel[1,], James M.S. Wason[1,2,***], and Paul J. Newcombe[1,****]**

[1]MRC Biostatistics Unit, University of Cambridge, Cambridge CB2 0SR, U.K.

[2]Population Health Sciences Institute, Newcastle University, Newcastle upon Tyne NE2 4BN, U.K.

*email: jixiong.wang@mrc-bsu.cam.ac.uk

**email: ashish.patel@mrc-bsu.cam.ac.uk

***email: james.wason@mrc-bsu.cam.ac.uk

****email: paul.newcombe@mrc-bsu.cam.ac.uk

This paper has been submitted for consideration for publication in *Biometrics*

Web Appendix A: Alternative Family-Wise Error Rate Controlling Methods

In a multiple testing setting, given the desired overall significance level $\overline{\alpha}$, a Bonferroni correction requires an adjusted significance level for each individual test to be $\alpha = \overline{\alpha}/m$, where m is the total number of hypothesis tests. This correction can control the overall family-wise error rate under $m\alpha = \overline{\alpha}$, which follows immediately from Boole's inequality, free of dependence and distributional assumptions. Additionally, the hypotheses may be tested at any other pre-specified combination of levels that add up to $\overline{\alpha}$. This technique is known as "alpha spending", in the context of feature selection, funneling power into features we are more interested in.

The Šidák correction works under the assumption of independence between individual tests, by solving the equation $\alpha = 1-(1-\overline{\alpha})^{1/m}$. The Šidák Correction is conservative when tests are positively dependent. In contrast, the correction can be liberal for tests that are negatively dependent. We can express $(1-\overline{\alpha})^{1/m}$ as its Taylor expansion around $\overline{\alpha} = 0$ and write down the function as

$$\alpha = 1 - (1 - \frac{1}{m}\overline{\alpha} + \frac{1-m}{2m^2}\overline{\alpha}^2 - \cdots) = \frac{\overline{\alpha}}{m} - \frac{m-1}{2m^2}\overline{\alpha}^2 + \cdots$$

This only differs from the Bonferroni adjusted significance level $\overline{\alpha}/m$ with $O(\overline{\alpha}^2/m)$ when m is large. For example, with $\overline{\alpha} = 0.05$ and $m = 10,000$, the Šidák corrected significance level is approximately 0.00000513 which is only slightly less stringent than 0.000005 used by the Bonferroni correction.

A more complex step-down procedure (Holm-Bonferroni procedure) which is uniformly more powerful than the Bonferroni correction and controls the family-wise error rate under $\overline{\alpha}$ is described as below:

Step 1: Order the p values of the m hypotheses in ascending order: $p_{(1)}, p_{(2)}, ..., p_{(m)}$.

Step 2: For a given significance level $\overline{\alpha}$, find the minimal index k such that $p_{(k)} > \overline{\alpha}/(m+1-k)$.

Step 3: Reject the first $k-1$ null hypotheses $H_{(1)}, ..., H_{(k-1)}$ and accept $H_{(k)}, ..., H_{(m)}$.

Then we consider the scenario when the false null hypotheses are sparse, e.g. m_0, the number of biomarkers with true biomarker-treatment interactions, is small compared with m. Let m_1 be the number of rejected true null hypotheses, then the total number of rejections $k-1$ yields

$$k - 1 \leqslant m_0 + m_1 \approx m_0 + (m - m_0)\overline{\alpha} = m_0(1 - \overline{\alpha}) + m\overline{\alpha} \approx m\overline{\alpha}$$

The first approximate equality sign $\approx$ follows from the fact that m_1 is close to its expectation $E(m_1) = (m - m_0)\overline{\alpha}$ with high possibility, because when the majority of biomarkers are in low linkage disequilibrium with each other, $var(m_1) \approx m_1\overline{\alpha}(1-\overline{\alpha})$ is small relative to m_1. The second $\approx$ follows from the $m_0 \ll m$ assumption. Thus, the hypotheses are essentially compared with the significance levels $\overline{\alpha}/m, \overline{\alpha}/(m-1), \ldots, \overline{\alpha}/(m+1-k) \approx \overline{\alpha}/\{m(1-\overline{\alpha})\}$. The Taylor series of $\overline{\alpha}/\{m(1-\overline{\alpha})\}$ around $\overline{\alpha} = 0$ is

$$\frac{\overline{\alpha}}{m(1-\overline{\alpha})} = \frac{\overline{\alpha}}{m} + \frac{\overline{\alpha}^2}{m} + \cdots$$

This again differs from the Bonferroni adjusted one $\overline{\alpha}/m$ with $O(\overline{\alpha}^2/m)$.

In a very similar method, the Hochberg procedure, rejection of $H_1, ..., H_k$ is made after finding the maximal index k such that $p_k \leqslant \overline{\alpha}/(m+1-k)$. This method is more powerful than the Holm-Bonferroni procedure, but requires the hypotheses are independent or under certain forms of positive dependence. In a similar manner to the argument for Holm-Bonferroni, one can demonstrate that the improvement by applying this method is subtle when biomarker-treatment interactions are sparse.

Web Appendix B: Discussion of Case-Only Style Interaction Tests in Randomized Clinical Trials

For the gene-environment interaction study, case-only tests were proposed for analysis under the assumptions: 1) the environmental factor and genetic variants occur independently; 2) the disease is rare and has a binary endpoint. In the context of analyzing biomarker-treatment interactions in randomized clinical trials, we consider a case-only style design where cases are defined by the trial outcome. This type of tests correspond to the following logistic regression model:

$$logit\{E(T_i \mid X_{ij}, R_i = 1)\} = \gamma_{0_j} + \gamma_{X_j} X_{ij}$$

where the biomarker-treatment independence condition and the rareness of response events are assumed, i.e. $pr(R_i = 1|X_{ij}) \approx 0$. Under such conditions, it can be showed that the estimator of γ_{X_j} is a consistent estimator of the interaction coefficient estimator in the standard interaction test. When the response is not rare, the estimator is biased in either a positive or negative direction depending on the sign of the true interaction effect.

Generally the rare response condition does not hold in randomized clinical trials. Only very large trials are powered to show a treatment effect for a rare response outcome. This prevents applying such approaches to finding biomarker-treatment interactions in principle. We note also that the additional power gained by a case-only analysis compared with a standard interaction test comes from the current data set with cases oversampled relative to their prevalence in the population. Given a disease is rare, a case-only test is equivalent to a comparable standard test with infinitely many controls. Thus, although biomarker-treatment independence is guaranteed in randomized clinical trials, case-only tests cannot outperform standard tests, as the trial population represents the entire dataset that has been randomized and thus cases (corresponding to responders) are not "oversampled". Based on the above argument, other proposals, e.g. empirical Bayes (Mukherjee and Chatterjee, 2008) and Bayes

model averaging (Li and Conti, 2008), involving combining case-only and standard tests, are of limited use in the settings we are interested, because no additional information can be provided even if the biomarker-treatment dependence is not a concern for us.

Web Appendix C: Proof of Lemma 1

The proof of Theorem 3 in Fu (2003) gave

$$n^{1/2}(\hat{\boldsymbol{\delta}}^{\lambda} - \boldsymbol{\delta}) = -\{\frac{1}{n}\nabla_{\boldsymbol{\delta}\boldsymbol{\delta}^T} L_n(\boldsymbol{\delta}) + \frac{2\lambda_n}{n}\}^{-1}\{\frac{1}{n^{1/2}}\nabla_{\boldsymbol{\delta}} L_n(\boldsymbol{\delta}) + \frac{2\lambda_n \boldsymbol{\delta}}{n^{1/2}}\}$$

for the ridge estimator. Under regularity conditions described in Van der Vaart (2000, p. 51-52): $\nabla_{\boldsymbol{\delta}} L_n(\boldsymbol{\delta})/n^{1/2}$ is asymptotically normal with a mean 0 and a finite variance σ^2 by the central limit theorem; $\nabla_{\boldsymbol{\delta}\boldsymbol{\delta}^T} L_n(\boldsymbol{\delta})/n$ converges in probability to $\boldsymbol{\Sigma}$ by the law of large numbers. When $\lambda_n = O(n^{1/2})$, $2\lambda_n/n$ vanishes and $2\lambda_n\boldsymbol{\delta}/n^{1/2}$ goes to $2\lambda_0\boldsymbol{\delta}$. Thus,

$$n^{1/2}(\hat{\boldsymbol{\delta}}^{\lambda} - \boldsymbol{\delta}) \rightarrow \mathcal{N}(-2\lambda_0\boldsymbol{\Sigma}^{-1}\boldsymbol{\delta}, \sigma^2\boldsymbol{\Sigma}^{-1})$$

A similar result for ridge regression can be immediately derived by Theorem 2 in Knight et al. (2000). When $\lambda_0 = 0$, this reduces to a well known result for the multivariate regression estimator without regularization

$$n^{1/2}(\hat{\boldsymbol{\delta}}^{0} - \boldsymbol{\delta}) \rightarrow \mathcal{N}(0, \sigma^2\boldsymbol{\Sigma}^{-1})$$

Web Appendix D: Proof of Corollary 1

Based on Lemma 1, we know the distribution of $n^{1/2}(\hat{\boldsymbol{\delta}}^{\lambda} - \boldsymbol{\delta})$ differs from that of $n^{1/2}(\hat{\boldsymbol{\delta}}^{0} - \boldsymbol{\delta})$ asymptotically only with a constant. Along with Theorem 1, the following holds immediately

$$cov\{n^{1/2}(\hat{\delta}^{\lambda}_{X_j} - \delta_{X_j}), n^{1/2}(\hat{\beta}_{X_j\times T} - \beta_{X_j\times T})\} \rightarrow 0$$

in probability.

Web Appendix E: Additional Simulation Results

In Web Fig. 1(a) and (b), we provide the graphs of family-wise error rates corresponding to the two scenarios shown in Fig. 1(a) and (b) from the paper. It is shown that all the four procedures control the family-wise error rates around or under the desired level 0.05.

[Figure 1 about here.]

In Web Fig. 1(c), we compared power of the different screening strategies while varying the proportion of explained variation by the true model. Specifically, we changed the standard deviation of the normal distribution from which ε_i was drawn from. For this exploration, biomarkers were set to be correlated at 0.6 and the sample size to $1,500$. Web Fig. 1(c) shows that when the true model explains either a low or high proportion of the variance, all the methods tend to perform similarly to each other. In the wide spectrum between the two extremes, the comparison is rather consistent: the penalized regression screening strategy performs best, followed by the two univariate screening procedures, with the single-step interaction test always resulting in the lowest power.

In Web Fig. 1(d), we changed only the correlation among biomarkers to examine how it affects the power comparison of these screening methods. It is shown that with the increasing correlation, power of the univariate screening procedure (rank) reduces and the benefit using the penalized regression screening strategy increases.

Web Appendix F: Additional Data Application Results

Correlation plots of covariates in the two real data applications are provided. It is shown in Web Fig. 2(a), correlations among the covariates from the START trial data are generally low. In contrast, Web Fig. 2(b) shows the PREVAIL trial data have highly correlated covariates.

[Figure 2 about here.]

References

Fu, W. J. (2003). Penalized estimating equations. *Biometrics* **59,** 126–132.

Knight, K., Fu, W., et al. (2000). Asymptotics for lasso-type estimators. *The Annals of statistics* **28,** 1356–1378.

Li, D. and Conti, D. V. (2008). Detecting gene-environment interactions using a combined case-only and case-control approach. *American journal of epidemiology* **169,** 497–504.

Mukherjee, B. and Chatterjee, N. (2008). Exploiting gene-environment independence for analysis of case–control studies: an empirical bayes-type shrinkage estimator to trade-off between bias and efficiency. *Biometrics* **64,** 685–694.

Van der Vaart, A. W. (2000). *Asymptotic statistics*, volume 3. Cambridge university press.

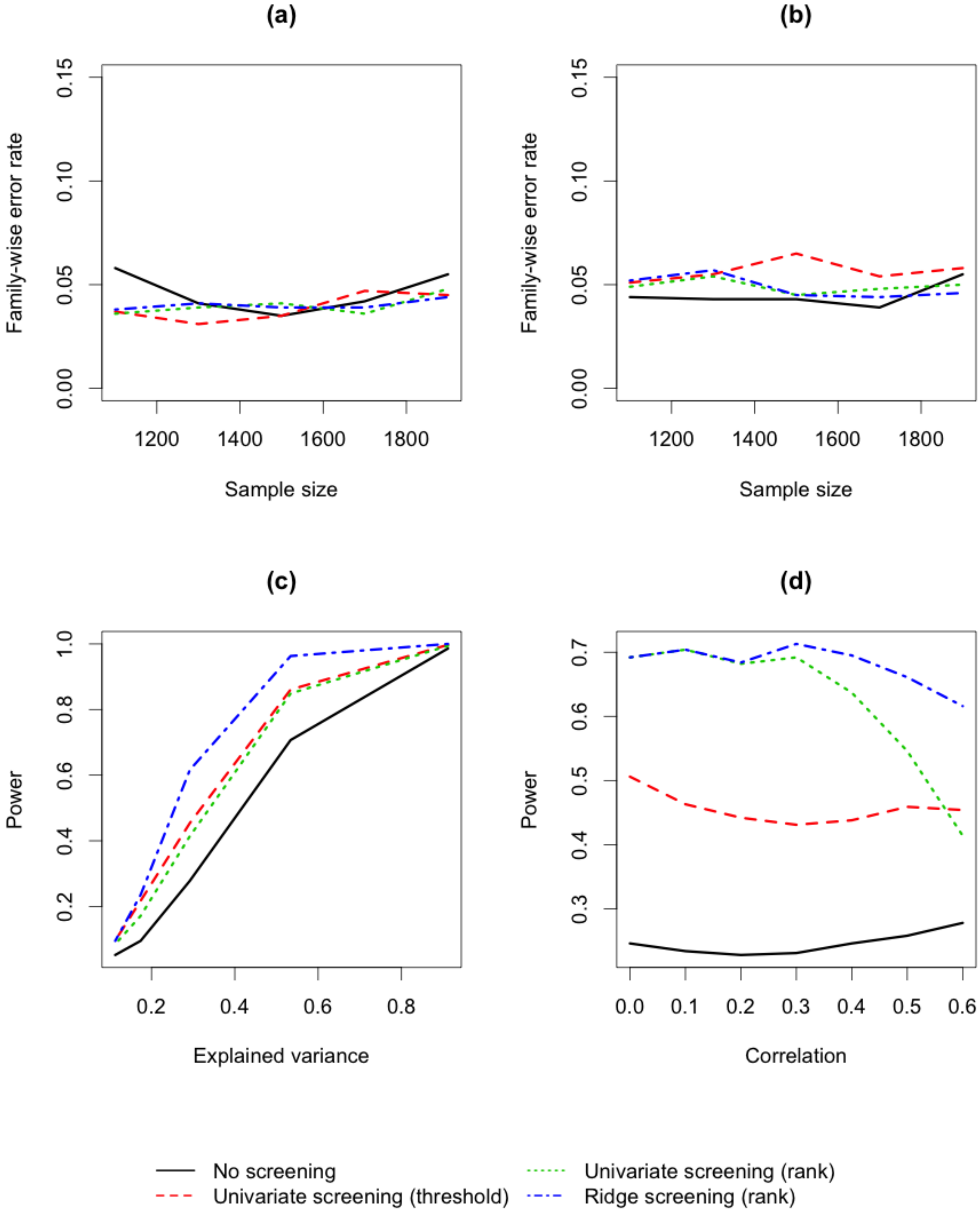

Web Figure 1: Comparison of two-stage interaction tests with different screening testing procedures. Four methods compared: univariate screening (threshold) (long dashes), univariate screening (rank) (short dashes), ridge screening (rank) (dot-dash), and no screening (solid). The four panels represent: (a) highly correlated biomarkers ($\rho = 0.6$), (b) independent biomarkers ($\rho = 0$), (c) highly correlated biomarkers ($\rho = 0.6$, sample size of $1,500$), changing the standard deviation of the normal distribution from which ε_i was drawn and consequently the variance explained in the outcome, (d) changing the biomarker-biomarker correlation.

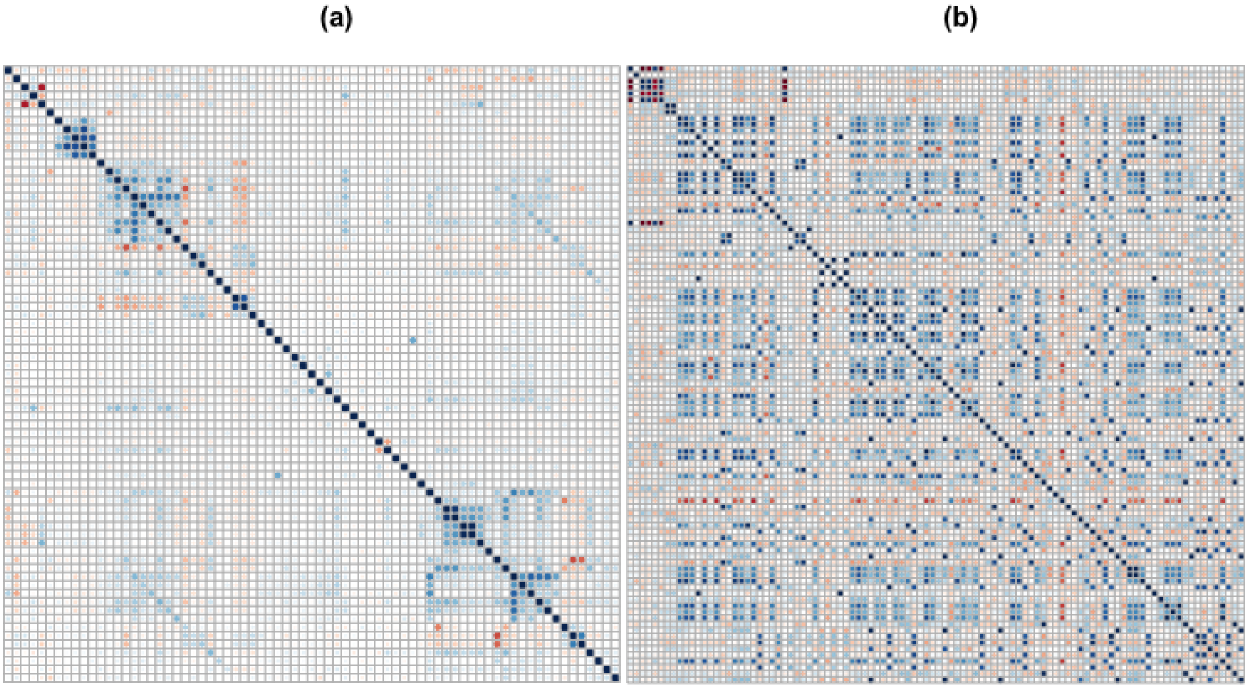

Web Figure 2: Correlation heat maps (blue: positive correlation; white: zero correlation; red: negative correlation) of: (a) 75 covariates from the START trial data; (b) 100 randomly selected covariates from the PREVAIL trial data.
