# Supplementary material for: Two-stage penalized regression screening to detect biomarker-treatment interactions in randomized clinical trials
Source: Biometrics. Author manuscript; Available in PMC 2022 Nov 27. (PMC7613856; doi:10.1111/biom.13424)
Supplement: Appendix [file EMS157113-supplement-Appendix.pdf]

SUPPORTING INFORMATION

Web Appendices referenced in Sections 2 and 3, and the R code for simulation studies and data applications are available with this paper at the Biometrics website on Wiley Online Library.

15410420, 2022, 1, Downloaded from https://onlinelibrary.wiley.com/doi/10.1111/biom.13424 by Test, Wiley Online Library on [15/11/2022]. See the Terms and Conditions (https://onlinelibrary.wiley.com/terms-and-conditions) on Wiley Online Library for rules of use; OA articles are governed by the applicable Creative Commons License

How to cite this article: Wang J, Patel A, Wason JMS, Newcombe PJ. Two-stage penalized regression screening to detect biomarker–treatment interactions in randomized clinical trials. *Biometrics*. 2022;78:141–150. https://doi.org/10.1111/biom.13424

APPENDIX A

A.1 | Proof of Theorem 1

Based on the unified approach to proving between-stage asymptotic independence by Dai *et al.* (2012), we need to evaluate the covariance matrix $\boldsymbol{A}_1^{-1}\boldsymbol{B}\boldsymbol{A}_2^{-1}$, where

$$\boldsymbol{A}_1 = E[(\boldsymbol{X}_i\boldsymbol{X}_i^T)\{Y_i - E(Y_i \mid \boldsymbol{X}_i)\}^2]$$

$$\boldsymbol{B} = E[(\boldsymbol{X}_i\boldsymbol{V}_{ij}^T)\{Y_i - E(Y_i \mid \boldsymbol{X}_i)\}\{Y_i - E(Y_i \mid \boldsymbol{V}_{ij})\}]$$

$$\boldsymbol{A}_2 = E[(\boldsymbol{V}_{ij}\boldsymbol{V}_{ij}^T)\{Y_i - E(Y_i \mid \boldsymbol{V}_{ij})\}^2].$$

We simplify the expression of $\boldsymbol{B}$ as

$$\begin{aligned}\boldsymbol{B} &= E[(\boldsymbol{X}_i\boldsymbol{V}_{ij}^T)\{Y_i^2 - Y_iE(Y_i \mid \boldsymbol{X}_i) - Y_iE(Y_i \mid \boldsymbol{V}_{ij}) \\ &\quad + E(Y_i \mid \boldsymbol{X}_i)E(Y_i \mid \boldsymbol{V}_{ij})\}] \\ &= E[(\boldsymbol{X}_i\boldsymbol{V}_{ij}^T)E\{Y_i^2 - Y_iE(Y_i \mid \boldsymbol{X}_i) - Y_iE(Y_i \mid \boldsymbol{V}_{ij}) \\ &\quad + E(Y_i \mid \boldsymbol{X}_i)E(Y_i \mid \boldsymbol{V}_{ij}) \mid \boldsymbol{X}_i\}] \\ &= E(\boldsymbol{X}_i\boldsymbol{V}_{ij}^T)\text{var}(Y_i \mid \boldsymbol{X}_i)\end{aligned}$$

which uses the law of iterated expectations, the fact that $\boldsymbol{X}_i$ includes $\boldsymbol{V}_{ij}$ under the null hypothesis $\beta_{X_j \times T} = 0$, and assumes homogeneity of variance, that is $\text{var}(Y_i \mid \boldsymbol{X}_i)$ is a constant.

Similarly, we have $\boldsymbol{A}_1 = E(\boldsymbol{X}_i\boldsymbol{X}_i^T)\text{var}(Y_i \mid \boldsymbol{X}_i)$ and $\boldsymbol{A}_2 = E(\boldsymbol{V}_{ij}\boldsymbol{V}_{ij}^T)\text{var}(Y_i \mid \boldsymbol{V}_{ij})$. Thus,

$$\boldsymbol{A}_1^{-1}\boldsymbol{B}\boldsymbol{A}_2^{-1} \propto E(\boldsymbol{X}_i\boldsymbol{X}_i^T)^{-1}E(\boldsymbol{X}_i\boldsymbol{V}_{ij}^T)E(\boldsymbol{V}_{ij}\boldsymbol{V}_{ij}^T)^{-1}.$$

We consider the second and the third terms:

$$\underset{(m+1)\times 3}{E(\boldsymbol{X}_i\boldsymbol{V}_{ij}^T)} = \begin{Bmatrix} E(T_iX_{ij}) & E(T_i^2) & E(T_i^2X_{ij}) \\ E(X_{i1}X_{ij}) & E(T_iX_{i1}) & E(T_iX_{i1}X_{ij}) \\ \vdots & \vdots & \vdots \\ E(X_{im}X_{ij}) & E(T_iX_{im}) & E(T_iX_{im}X_{ij}) \end{Bmatrix}$$

$$\underset{3\times 3}{E(\boldsymbol{V}_{ij}\boldsymbol{V}_{ij}^T)^{-1}} = \frac{1}{det\{E(\boldsymbol{V}_{ij}\boldsymbol{V}_{ij}^T)\}}$$

$$\times\begin{Bmatrix} \cdot & \cdot & E(T_iX_{ij})E(T_i^2X_{ij}) - E(T_i^2)E(T_iX_{ij}^2) \\ \cdot & \cdot & E(T_iX_{ij})E(T_iX_{ij}^2) - E(X_{ij}^2)E(T_i^2X_{ij}) \\ \cdot & \cdot & E(X_{ij}^2)E(T_i^2) - E(T_iX_{ij})^2 \end{Bmatrix}.$$

Thus, for the $(m+1)\times 3$ matrix $E(\boldsymbol{X}_i\boldsymbol{V}_{ij}^T)E(\boldsymbol{V}_{ij}\boldsymbol{V}_{ij}^T)^{-1}$, the $(k+1)$th element $(k = 1, \ldots, m)$ of the last column is proportional to

$$\begin{aligned}&\{E(X_{ik}X_{ij}), \quad E(T_iX_{ik}), \quad E(T_iX_{ik}X_{ij})\} \\ &\times\begin{Bmatrix} E(T_iX_{ij})E(T_i^2X_{ij}) - E(T_i^2)E(T_iX_{ij}^2) \\ E(T_iX_{ij})E(T_iX_{ij}^2) - E(X_{ij}^2)E(T_i^2X_{ij}) \\ E(X_{ij}^2)E(T_i^2) - E(T_iX_{ij})^2 \end{Bmatrix} \\ &= E(T_i)\text{var}(T_i)E(X_{ij}) \\ &\quad \times\{E(X_{ik}X_{ij})E(X_{ij}) - E(X_{ik})E(X_{ij}^2)\} = 0,\end{aligned}$$

which uses the independence between T_i and X_{ij} and the assumption $E(T_i) = 0$ or $E(X_{ij}) = 0$. Similarly, the first element of the last column is also zero.

Premultiplying $E(\boldsymbol{X}_i\boldsymbol{V}_{ij}^T)E(\boldsymbol{V}_{ij}\boldsymbol{V}_{ij}^T)^{-1}$ by $E(\boldsymbol{X}_i\boldsymbol{X}_i^T)^{-1}$ completes the covariance matrix, the last column of which are all zeros. Thus, for any $j = 1, \ldots, m$, we have $\text{cov}\{n^{1/2}(\hat{\delta}_{X_j}^0 - \delta_{X_j}), n^{1/2}(\hat{\beta}_{X_j\times T} - \beta_{X_j\times T})\} \to 0$ in probability.

15410420, 2022, 1, Downloaded from https://onlinelibrary.wiley.com/doi/10.1111/biom.13424 by Test, Wiley Online Library on [15/11/2022]. See the Terms and Conditions (https://onlinelibrary.wiley.com/terms-and-conditions) on Wiley Online Library for rules of use; OA articles are governed by the applicable Creative Commons License
